# Are dogs with hip dysplasia in less pain after total hip replacement than femoral head ostectomy?

**DOI:** 10.18849/ve.v7i1.388

**Published:** 2022-01-07

**Authors:** Erica Rehnblom+, Wanda J. Gordon-Evans+

**Keywords:** CANINE, HIP DYSPLASIA, FEMORAL HEAD OSTECTOMY, FEMORAL HEAD OSTEOTOMY, FEMORAL HEAD EXCISION, FEMORAL NECK EXCISION, EXCISION ARTHROPLASTY, TOTAL HIP REPLACEMENT, TOTAL HIP ARTHROPLASTY

## Abstract

PICO question

In large breed juvenile dogs with hip dysplasia and radiographic bilateral osteoarthritis, is a total hip replacement superior/inferior/or equivalent to bilateral femoral head ostectomy at reducing the severity of long-term hip pain?

Clinical bottom line

Category of research question

Treatment

The number and type of study designs reviewed

Twelve papers were critically appraised. One paper was a systematic review. Six papers were prospective case series. Five papers were retrospective case series

Strength of evidence

Weak

Outcomes reported

Besides one systematic review, there are no other studies available that directly compare pain reduction with total hip replacement and femoral head ostectomy for the treatment of hip dysplasia in large breed juvenile dogs with radiographic evidence of secondary osteoarthritis. In one study, 12/12 (100%) of owners that responded to an owner outcome questionnaire reported no hip pain with femoral head and neck ostectomy. In this study, owners assessed pain based on activity level of the dog (running, playing, jumping, using stairs normally), gait abnormalities (only when running or after strenuous exercise), and duration of postoperative medications. In eight studies, 91–100% of cases had no hip pain with total hip replacement reported via clinical examination and/or owner outcome questionnaire

Conclusion

There is evidence suggesting that both total hip replacement and femoral head ostectomy may be capable of reducing long-term pain as a result of osteoarthritis, secondary to hip dysplasia, however, based on the current literature, it is challenging to say whether total hip replacement is superior to femoral head and neck ostectomy at reducing long-term hip pain. It is important to recognise that other factors considered as outcomes (i.e. range of motion, ground reaction forces, force-plate analysis, etc.) may contribute to differing outcomes overall for total hip replacement vs femoral head ostectomy, but this paper focused specifically on pain. While there is a systematic review that provides evidence supporting that total hip replacement is superior at returning dogs to normal function, evaluating return to normal function was not the focus of this Knowledge Summary

## How to apply this evidence in practice

The application of evidence into practice should take into account multiple factors, not limited to: individual clinical expertise, patient’s circumstances and owners’ values, country, location or clinic where you work, the individual case in front of you, the availability of therapies and resources.

Knowledge Summaries are a resource to help reinforce or inform decision making. They do not override the responsibility or judgement of the practitioner to do what is best for the animal in their care.

## Clinical scenario

You diagnosed a 1 year old female Golden Retriever with bilateral hip dysplasia and persistent hip pain despite appropriate non-surgical management. She shifts her weight forward, bunny hops and has radiographic osteoarthritis. You recommend a total hip replacement or bilateral femoral head ostectomy. The owner wants to know if one option is better at reducing the severity of long-term hip pain.

## The evidence

There is only one prospective case series that directly addresses the present clinical question, however the included population size is very small. Of the literature that solely addresses total hip replacement outcome, five studies are prospective case series and five are retrospective case series. Of the literature that solely addresses femoral head ostectomy outcome, one study is a retrospective case series. There is one systematic review that addresses both total hip replacement and femoral head ostectomy, however return to normal function was their main outcome measurement rather than pain. Overall, because there is only one paper with a small population size directly addressing the present clinical question, the other included literature does not directly compare total hip replacement and femoral head ostectomy for long-term hip pain reduction, and because the current studies all use differing outcome measures and thus a direct comparison cannot be made, it is impossible to draw a meaningful conclusion as to which procedure is more efficacious at reducing long-term hip pain by comparing these studies.

THR – total hip replacement

FHO – femoral head ostectomy

LOAD questionnaire – Liverpool Osteoarthritis in Dogs questionnaire

GRF – ground reaction force

## Summary of the evidence

**Dueland et al. (1977):** 

Population:	17 dogs with degenerative joint disease secondary to hip dysplasia that underwent either THR and/or FHO, and four unaffected dogs as controls.
Sample size:	21 dogs.
Intervention details:	17/29 dogs were selected for this study that were >25 kg, had radiographic evidence of dysplasia (except for the four unaffected dogs), had clinically successful THR and/or FHO, and had an analysis done after a minimum of 1 year postoperatively Group 1 (4/17): normal, unoperated dogs. Group 2 (4/17): dogs with unoperated hip vs THR. Group 3 (5/17): dogs with one THR and one FHO. Group 4 (4/17): dogs with bilateral THR. Force-plate analysis was done to compare vertical and horizontal forces. Speed of dog running was obtained by running them over a known distance and timing this. A linear relationship between velocity and force was established using least squares curve fitting technique.
Study design:	Prospective case series.
Outcome studied:	Objective: force-plate analysis. Subjective: clinical evaluation of gait and lameness.
Main Findings (relevant to PICO question)	When pain is reduced, the landing forces of the limb are increased. Therefore, if a higher vertical force is apparent on one side in an individual, one should assume that this equals more weight bearing and better performance on that side. Group 1: normal gait and comparable values between right and left hips in vertical forces. Group 2: in 3/4 dogs, more vertical force was apparent on the normal, unoperated side. Clinically, all four dogs walked and ran within normal limits. Group 3: all five dogs showed normal gait, 3/5 showed higher vertical force on the FHO side. Group 4: all four dogs showed little clinical difference between the two hips, 3/4 dogs showed comparable vertical force between the two hips. Successful THR may functionally approach a normal hip or improve a dysplastic hip. Successful FHO is still a valuable surgical treatment option which may equal or surpass THR clinically and biomechanically.
Limitations:	Small study population. Case series. Clinicians were unblinded during lameness evaluation. Non-randomised study population.

**Olmstead et al. (1983):** 

Population:	Dogs with hip disease (predominantly hip dysplasia) that underwent THR at The Ohio State University Veterinary Teaching Hospital (Columbus, Ohio, US) and Berwyn Veterinary Associates Hospital (Berwyn, Illinois, US).
Sample size:	190 dogs.
Intervention details:	Between August 1976 and July 1981, 221 THRs were performed in 190 dogs. Disabling hip dysplasia was the indication for THR in 182/221 procedures. Physicals, radiographic examination, and owner histories were used in pre- and yearly postoperative patient evaluation. Dogs were evaluated before and yearly thereafter until the dog died, prosthesis was removed, or the owner could no longer be contacted.
Study design:	Prospective case series.
Outcome studied:	Subjective: clinical evaluation of lameness, owner questionnaire.
Main Findings (relevant to PICO question)	149 THRs were still being monitored by January 1982, and of these cases, 136/149 (91.2%) had satisfactory results when function could be evaluated. Owners reported satisfactory results as an increase in muscle mass of the operated leg, and elimination of signs of pain. All 152 dogs with hip dysplasia included in the study were bilaterally affected, and unilateral replacement was adequate for 122 of the dogs. It is assumed that THR on one side allowed the dog to shift more of the weight bearing burden in that direction, relieving pressure on the dysplastic hip. Only when a complication was encountered was there a risk that function would be unsatisfactory. 46/221 (20.8%) procedures had complications, and 27/46 (58.7%) ultimately achieved satisfactory function after revision.
Limitations:	Follow-up could not be completed on the entire population. Case series. Evaluation of function by examiner and owner is very subjective.

**Parker et al. (1984):** 

Population:	Dogs with degenerative joint disease secondary to hip dysplasia underwent THR using a Richards Canine II large size prosthesis.
Sample size:	20 dogs.
Intervention details:	17 dogs underwent unilateral THR only, and three dogs underwent bilateral THR (23 THRs) and were evaluated before and at 3 month intervals for 1 year postoperatively. All cases underwent preoperative evaluation which included a history, complete physical examination, neurological examination, complete blood count, chemistry panel, and radiographs. All cases had follow-up which consisted of direct observation or an owner telephone interview. The grading system for the affected leg(s) was adapted from Gendreau & Cawley (1977) and consisted of excellent (total weight bearing), good (slight lameness or gait abnormality), fair (noticeable lameness and may be non-weight bearing when running), and poor (severe lameness and may be non-weight bearing at all times.
Study design:	Prospective case series.
Outcome studied:	Subjective: clinical evaluation of lameness, owner telephone interview.
Main Findings (relevant to PICO question)	18/23 (78%) of cases were graded as good or excellent. One patient required revision surgery to achieve a good outcome. 5/23 (22%) of cases had poor or fair outcomes, and all developed permanent post-operative complications or had progression of undiagnosed pre-operative problems (degenerative myelopathy).
Limitations:	Small study population. Case series. Clinicians were unblinded during lameness evaluation. Subjective owner telephone interviews used to determine outcome. Study included some elderly dogs.

**Massat et al. (1994):** 

Population:	Dogs that underwent cemented THR.
Sample size:	84 dogs.
Intervention details:	Medical records were reviewed for all dogs that underwent THR from April 1986 to February 1992. Dogs were included in the study if negative pressure had been applied to the medullary cavity of the ilium during cementing of the acetabular component. 96 cases were included. 88 cases were performed secondary to hip dysplasia. Mean age 6.5 years (range: 10 months–13 years) 58 of the cases were evaluated by one of the authors or a referring veterinarian. Mean follow-up time was 25.1 months (range: 6–70 months). The remaining 38 cases were evaluated via owner assessment outcome. Mean follow-up time for these cases was 26.7 months (range: 3–75 months).
Study design:	Retrospective case series.
Outcome studied:	Objective: thigh circumference. Subjective: clinical evaluation, owner assessment outcome.
Main Findings (relevant to PICO question)	42/58 cases clinically evaluated were graded as excellent, 15 were graded as good. Limb function was graded as poor for one. 34 of the owner assessment outcome cases were graded as excellent, one as good, and three as fair. All four dogs with fair or poor results had complications which necessitated implant removal. Six cases with excellent clinical outcome were selected for GRF analysis. Two dogs with contralateral degenerative joint disease had significantly greater vertical force in the THR limb than for the contralateral limb. The other four dogs had bilateral THR or unilateral THR with normal contralateral coxofemoral joint. No significant difference in peak vertical force in the hindlimbs of these four dogs was detected. 8/84 dogs had complications. Four were corrected and hip function was eventually good or excellent. Implant was removed in the other four dogs, and final hip function was fair in three dogs and poor in one. Cemented THR is an effective treatment for disabling conditions of the coxofemoral joint in dogs.
Limitations:	Retrospective study design. Case series. Subjective owner assessment outcome. Not all cases were clinically evaluated. Two different THR systems were used. Thigh circumference was only measured in good or excellent results. Of the 6/84 dogs that underwent GRF testing, two had bilateral THR, two had unilateral THR with normal contralateral coxofemoral joint, and two had unilateral THR and had contralateral degenerative joint disease. This variation in disease states can introduce significant bias when analysing GRF.

**Budsberg et al. (1996):** 

Population:	Dogs with degenerative joint disease secondary to hip dysplasia underwent unilateral cemented THR.
Sample size:	16 dogs.
Intervention details:	Dogs underwent unilateral THR and were evaluated before and at 1, 3, 6, and 12 months post-operatively. Of 16 cases, 14 had ≥12 months follow-up consisting of clinical evaluation and GRF.
Study design:	Prospective case series.
Outcome studied:	Objective: GRF. Subjective: clinical evaluation of lameness.
Main Findings (relevant to PICO question)	GRF indicated significantly increased loading function of treated limb by 6 months postoperatively. Vertical impulse decreased in untreated limb over course of study, indicating that dogs were beginning to compensate by shifting function to the treated limb. Lameness scores for 14/16 dogs were available at 12 months and were 0.1 ± 0.3, and were available for 5/16 dogs at 24 months, which were all 0. A lameness score was graded from 0–4, with 0 being a normal gait and a 4 being non-weight bearing.
Limitations:	Small study population. Case series. Clinicians were unblinded during lameness evaluation. Only five dogs had follow-up to 24 months.

**Rawson et al. (2005):** 

Population:	Dogs referred to the Veterinary Specialists of South Florida for evaluation of hip dysplasia and simultaneous bilateral FHO.
Sample size:	15 dogs.
Intervention details:	Medical records were reviewed in all dogs from July 2000 to June 2003 that had radiographic evidence of bilateral osteoarthritis secondary to hip dysplasia and underwent simultaneous bilateral FHO. 12/15 dogs were evaluated via owner between 6–48 months postoperatively via telephone survey. Mean age at surgery 10.3 months (range: 6–25 months).
Study design:	Retrospective case series.
Outcome studied:	Subjective: owner outcome assessment (time to full recovery, evidence of pain, and overall satisfaction with the surgical procedure, concurrent orthopaedic problems). Objective: duration and current medications.
Main Findings (relevant to PICO question)	Five owners graded results as excellent, seven graded the results as good. No dogs had hip pain at time of follow-up. Seven dogs had slightly abnormal gait only when running or after strenuous exercise.
Limitations:	Retrospective study design. Case series. Wide range in follow-up time. Subjective owner outcome assessment. All dogs were offered follow-up examination and radiographs. These were only performed in four dogs. Only 12/15 (80%) of owners responded in a small study size.

**Guerrero et al. (2009):** 

Population:	Dogs with degenerative joint disease secondary to hip dysplasia underwent 2^nd^ generation Zurich cementless THR.
Sample size:	60 dogs.
Intervention details:	59/65 THRs were completed between April 2001 and September 2003 on dogs with hip dysplasia and secondary coxoarthrosis. Information obtained included breed, sex, body weight, date of surgery, indication for THR, operated side, surgical time, angles lateral opening (ALO), inclination of cup (AI), longest clinical and radiographic follow-up, intra- and postoperative complications, management of complications, and outcome. One surgeon performed all THRs. All dogs had postoperative evaluation which included clinical evaluation (pain on manipulation of hip joint, range of motion, muscle mass compared with contralateral leg, lameness) ≥6 months. Clinical evaluation was scored based on a previously reported scale, of excellent, good, fair, poor, or failed function. ** **
Study design:	Prospective case series.
Outcome studied:	Subjective: clinical evaluation of lameness.
Main Findings (relevant to PICO question)	60/65 THRs were considered to have excellent clinical outcome, three had good outcome, and two as failed outcome. High complication rates, 11/65 (17%), consisted predominantly of THR luxations, 7/65 (11%). After revision, a successful outcome was achieved in 63/65 (97%) of THRs.
Limitations:	Small study population. Case series. Clinicians were unblinded during lameness evaluation. Previously reported clinical evaluation scale was not disclosed.

**Gemmill et al. (2011):** 

Population:	Dogs undergoing hybrid THR using a cementless acetabular component and a cemented femoral component.
Sample size:	71 dogs.
Intervention details:	Dogs were selected for the study from December 2005 to July 2009 if they had debilitating hip disease unresponsive to conservative management and absence of concurrent medical, orthopaedic, or neurologic disease that would preclude surgery. Follow-up ≥ 6 months. Of 78 procedures, hip dysplasia was indicated in 68. Dogs were evaluated at 4 and 12 weeks, and 6–27 months by veterinarians. Owner outcome assessment completed 6–40 months postoperatively.
Study design:	Prospective case series.
Outcome studied:	Subjective: owner outcome assessment, pain upon manipulation by veterinarian.
Main Findings (relevant to PICO question)	Pain, lameness, and disability were reported to be mild in 3/78 (3.8%) cases and absent in 75/78 (96.2%) cases. 73/78 (94%) of cases were reported to have normal quality of life at owner assessment outcome. Of four cases that had postoperative complications, three were successfully revised.
Limitations:	Case series. Veterinarian evaluation was done by the vet who performed the procedure, thus bias may be introduced by multiple examiners. Subjective owner outcome assessment.

**Bergh et al. (2014):** 

Population:	Dogs with naturally occurring canine hip dysplasia that were treated with various surgical procedures.
Sample size:	848 dogs.
Intervention details:	Of 477 manuscripts, 17 met inclusion criteria. These were grouped based on surgical procedure (THR, triple pelvic osteotomy, juvenile pubic symphysiodesis, Chiari osteotomy/intertrochanteric osteotomy, and FHO) and level of evidence (I–V) relative to the study question. There were seven total hip replacement studies and one femoral head ostectomy study. Unilateral surgeries with >6 months follow-up were included. The outcome measurements included were orthopaedic exam, owner interview, visual gait observation, and force plate gait analysis depending on the study.
Study design:	Systematic literature review.
Outcome studied:	Subjective: orthopaedic examination, owner interview/questionnaire, and/or visual gait observation. Objective: force plate gait analysis.
Main Findings (relevant to PICO question)	THR consistently returned dogs to normal function. FHO did not consistently return dogs to normal function.
Limitations:	The review was in depth but the studies measured mostly subjective outcomes. All THR and FHO studies had low levels of evidence. Comparison between procedures was difficult due to varying outcome measurements. The review assesses functional outcome instead of strictly pain outcome. Three total hip replacement studies had level III evidence, four had level IV evidence. The only FHO study had level IV evidence.

**Fitzpatrick et al. (2014):** 

Population:	Skeletally immature dogs with hip dysplasia undergoing THR using BioMedtrix BFX^TM ^biologic fixation implants after unsatisfactory outcome with medical management.
Sample size:	20 dogs.
Intervention details:	Medical records were reviewed from November 2007 to June 2010 and dogs that had unsatisfactory outcome with medical management due to coxofemoral pain resulting from canine hip dysplasia that underwent unilateral cementless THR using BFX^TM^ implants were selected for the study. All dogs at time of surgery were 6–10 months of age. All dogs were available for follow-up immediately and 6 weeks postoperatively, 19 were available for long-term follow-up.
Study design:	Retrospective case series.
Outcome studied:	Subjective: owner outcome assessment via questionnaire at final follow-up (function, pain, and analgesia requirements), veterinary examination score (maximum possible score of 11, including lameness, musculature, pain, and range of motion) immediately, at 6 weeks, and at long-term follow-up (mean of 29.8 months, range: 12–48 months).
Main Findings (relevant to PICO question)	Mean veterinary examination score 0.8 (range: 0–4) at long-term follow-up. Pain was not elicited in any and normal range of motion was achieved in all dogs at long-term follow-up. Mean activity score (via owner questionnaire) 1.3 (from 0–5, 5 being the worst) for seven activities (walking, sitting, rising, running, climbing stairs, getting into the car, play or exercise).
Limitations:	Retrospective study design. Case series. Limited follow-up (mean of 29.8 months) despite young age of the population. Small study population. Lack of a control group. Subjective outcome measurements.

**Vezzoni et al. (2015):** 

Population:	Dogs that underwent Zürich cementless THR at the Clinica Veterinaria Vezzoni S.R.L, Cremona, Italy.
Sample size:	321 dogs.
Intervention details:	Records were reviewed from January 2002 to December 2007 and of 348 dogs and 479 Z-THAs, 321 dogs and 439 cases fit the inclusion criteria. This included 191 juvenile cases, 248 adult cases, and 118 bilateral cases treated for hip dysplasia. Dogs with a follow-up ≥ 2 years were included. Juvenile group ≤ 11 months old. Adult group ≥ 11 months old. Follow-up was done at 2, 6, and 12 months postoperatively, and yearly thereafter with physical examination. Any outcome other than a normally functioning hip was classified as a complication.
Study design:	Retrospective case series.
Outcome studied:	Objective: weight change after surgery Subjective: clinical examination including body condition score, muscle mass, range of motion, presence of any pain or discomfort during manipulation, and video recording of gait.
Main Findings (relevant to PICO question)	Adult group: 33/238 (14%) complications, 31 successfully revised, two explanted. 10 cases were not accounted for. Juvenile group: 39/191 (20%) complications, 37 successfully revised, two explanted. Difference in complication rate not statistically significant.
Limitations:	Retrospective study design. Case series. Subjective outcome measurements. Two authors received honoraria and one received donated implants for biomechanical studies.

**Bayer et al. (2019):** 

Population:	Dogs with hip dysplasia that underwent THR using a combined implant system consisting of INNOPLANT Screw Cup, KYON taper head, and Zürich cementless (Z-THR) stem.
Sample size:	12 dogs.
Intervention details:	Records were reviewed from March 2010 to March 2015 and 16 hybrid THR procedures were performed in 12 dogs. Follow-up examination was performed at 2, 6, and 12 months postoperatively. Follow-up ≥2 years. Mean age at surgery 31 months (range: 12–98 months).
Study design:	Retrospective case series.
Outcome studied:	Objective: owner-administered outcome LOAD assessment. Osteoarthritis was defined as mild for LOAD scores 0–10, moderate for 11–20, severe for 21–30, and extreme from 31–52. Subjective: clinical outcome examination including pain or discomfort upon manipulation of hip joint, range of motion, lameness grade (0–4), and thigh circumference compared to contralateral limb.
Main Findings (relevant to PICO question)	15 cases had full function and one had acceptable function at final follow-up. In dogs with full function, all had thigh circumference that was bilaterally symmetrical. Median LOAD score for procedures with full functional outcome was 5 (range: 3–11). Three major complications occurred in three cases, all had complete revision and resolution.
Limitations:	Retrospective study design. Case series. Small study population. One surgeon performed all procedures, however they had limited experience (the surgeon had performed 28 THA procedures previously, compared to the proposed initial learning curve of a minimum of 44 THA procedures), which may introduce surgeon-related complications. Subjective outcome measurements.

## Appraisal, application and reflection

After a thorough search of the literature, only one paper was found that specifically addresses the PICO question, and twelve papers were found that partially address it. Included in this Knowledge Summary is one systematic review, six prospective case series, and five retrospective case series. Unfortunately, other than the systematic analysis and one prospective case series with force-plate analysis and very small population size, none of the other studies directly compare THR and FHO, and differing outcome measures used throughout the studies make it difficult to draw a meaningful conclusion in regards to the clinical question.

The strongest evidence available comes from the systematic review (Bergh et al., 2014). Included in the systematic review are seven manuscripts regarding THR and one manuscript regarding FHO. In regards to the clinical question, THR consistently returned dogs to normal limb function while FHO did not consistently return dogs to normal limb function. However, it is important to note that while the systematic review represents the highest level of evidence in this study, it is not without limitations. The systematic review focused on limb functionality, which only indirectly relates to the present clinical question in regards to reducing the severity of long-term pain. It is also important to point out that the manuscripts evaluated in the systematic review used differing outcome measurements and had low levels of evidence, which means that their conclusion must be interpreted with caution.

One prospective case series (Gemmill et al., 2011) analysed 71 dogs for a total of 78 hybrid THR replacement procedures with a cementless acetabular component and a cemented femoral component with follow-up of ≥ 6 months. At time of owner outcome assessment, 75/78 (96%) of dogs were reported to have no hip pain. This subjective outcome measure of owner outcome assessment and lack of a control group weakens the evidence of this study. A second prospective case series (Budsberg et al., 1996) analysed 16 dogs for a total of 16 unilateral cemented total hip replacement procedures, 14 of which had follow-up ≥ 12 months. All dogs in this study had significantly increased GRF for loading function of the treated limb by 6 months postoperatively. Five dogs had follow-up at 24 months, and at this time all five dogs had lameness scores of 0. This study represents one of the few studies with objective outcome measurements in the form of GRF, and thus strengthens the evidence for THR reducing the severity of long-term hip pain as a result of hip dysplasia. A third prospective case series (Olmstead et al., 1983) analysed 221 THRs in 190 dogs performed predominantly as a result of hip dysplasia using physicals, radiographic examination, and owner histories in pre- and yearly post-operative patient evaluation. 136/149 (91.2%) of THRs had satisfactory function at time of follow-up suggesting that THR may reduce the severity of long-term hip pain as a result of hip dysplasia, however the subjective outcome of clinical evaluation and owner assessment weaken the evidence of this study. A fourth prospective case series (Parker et al., 1984) analysed 20 dogs with 23 THRs using a Richards Canine II large size prosthesis. Outcome evaluation consisted of direct observation and owner telephone outcome assessment. Through direct observation, THR outcome was graded using a grading system for the affected leg(s) that was adapted from Gendreau & Cawley (1977) and consisted of excellent (total weight bearing), good (slight lameness or gait abnormality), fair (noticeable lameness and may be non-weight bearing when running), and poor (severe lameness and may be non-weight bearing at all times. 18/23 (78%) of THRs had excellent or good outcome. Subjective observation and owner telephone assessment weaken the evidence that THRs provide excellent to good outcome for decreasing pain in dogs with hip dysplasia, however using a standardised grading system minimises subjective grading bias. A fifth prospective case series (Guerrero et al., 2009) anaylsed 60 dogs that underwent 65 THRs using 2^nd^ generation Zurich cementless THR. All dogs had post-operative evaluation which included clinical evaluation (pain on manipulation of hip joint, range of motion, muscle mass compared with contralateral leg, lameness) ≥ 6 months, and clinical evaluation was scored based on a previously reported scale, of excellent, good, fair, poor, or failed function. 60/65 (92.3%) of THRs were considered to have excellent clinical outcome. Using a standardised scale for clinical evaluation minimises subjective clinical evaluation, however the scale used was not reported, weakening the evidence supporting that THRs decrease long-term pain associated with THR. Finally, a sixth prospective case series (Dueland et al., 1977) analysed 21 dogs, 17 of which had degenerative joint disease secondary to hip dysplasia that underwent either THR and/or FHO, and four unaffected dogs as controls. Dogs were divided into four groups, group 1 was normal, unoperated dogs, group 2 was dogs with one unoperated hip vs one THR, group 3 was dogs with one THR and one FHO, and group 4 was dogs with bilateral THRs. Force-plate analysis was done to compare vertical and horizontal forces, and clinical evaluation of gait and lameness was also reported. Based on force-plate analysis, THR may functionally approach a normal hip or improve a dysplastic hip, and successful FHO may equal or surpass THR clinically and biomechanically. This study provides strong objective analysis by using force-plate technology suggesting that THR and FHO may improve gait, lameness, and force of operated limb. However, the sample size was incredibly low for each group (4–5 dogs) so a larger study should be performed before these results can be more widely accepted.

There was one retrospective case series (Bayer et al., 2019) that analysed 12 dogs for a total of 16 THR procedures using a combined implant system consisting of INNOPLANT Screw Cup, KYON taper head, and Zürich cementless (Z-THR) stem. These cases were evaluated with clinical examination and LOAD questionnaires performed by the owners. 15/16 cases had full function while 1/16 cases had acceptable function. Median LOAD score was 5 (range: 3–11). The LOAD score is a standardised tool for collecting data from owners, and in this case supported the subjective clinical examination findings and outcome determined at the final follow-up visit that THR is efficacious at reducing the severity of long-term hip pain associated with hip dysplasia. Although the retrospective and subjective outcome measures weaken the evidence of this study, this is the only retrospective case study out of five that used a standardised owner outcome questionnaire.

Of the three remaining retrospective case series regarding THR, one study (Vezzoni et al., 2015) analysed 439 Zürich cementless THR procedures and all dogs except for four had normal clinical outcome via clinical examination at their 12 month follow-up, and the remaining two studies (Massat et al., 1994; and Fitzpatrick et al., 2014) used both clinical examination and owner assessment outcome as outcome measurements, and found 84/88 cases had good or excellent outcomes, and 20/20 cases had no pain upon clinical examination respectively.

There were only three studies for FHO outcome that related to the present clinical question. The first (Rawson et al., 2005) as discussed above, found that all dogs had no hip pain at time of follow-up according to owner questionnaire. The second study (Ganesh et al., 2017) was unavailable and thus is not included in the answering of the present clinical question. The third study (Dueland et al., 1977) used force-plate analysis to determine that successful FHO may improve gait, lameness, and force of operated limb, which may be interpreted as decreased pain, however the study population was very small. Unfortunately, FHO studies are lacking, which contributes to the difficulty in answering the present clinical question. Two studies (Off & Matis et al., 2010; and Duff et al., 1977) discussing FHO outcome were not included in this Knowledge Summary due to Legg-Calvé Perthes disease being the predominant indication for surgery rather than hip dysplasia, presenting the possibility that femoral head necrosis may affect FHO outcome differently than FHO outcome due to hip dysplasia.

From the available data, it is possible that both THR and FHO may reduce the severity of long-term pain as a result of osteoarthritis secondary to hip dysplasia given the owner assessment outcomes and clinical evaluation outcomes in the aforementioned studies. However, given the differing outcome measurements between studies such as subjective non-standardised owner assessment outcomes, subjective clinical evaluation, GRF, and force-plate analysis it is impossible to compare one study to another. It is also important to mention varying postoperative pain management and physical rehabilitation not only between different studies but between individual patients in a given study as an additional variable influencing outcome and ultimately long-term pain associated with FHO and THR. It is also important to note that there are several more studies analysing THR than FHO, which provides an unequal amount of evidence towards the success of THR at reducing long-term pain associated with hip dysplasia. From the evidence, it appears that THR provides a successful outcome in many cases, however it is not evident whether THR provides a superior/inferior/or equivalent outcome compared to FHO given the lack of studies analysing FHO. In order to definitively answer the present clinical question, a prospective, randomised clinical trial, with pre-determined standardised outcome measurements, comparing THR treated dogs to FHO treated dogs with naturally occurring hip dysplasia would be necessary.

Two papers (Vezzoni et al., 2015; and Bayer et al., 2019) were not indexed by either database using the below search terms, however both papers were deemed applicable based on reference checking. This represents an inherent bias of the chosen databases, and represents a limitation of this Knowledge Summary.

## Methodology Section

**Search Strategy:** 

Databases searched and dates covered:	CAB Abstracts on OVID Platform; 1973–Aug 2021 PubMed on NCBI Platform; 1972–Aug 2021
Search strategy:	CAB Abstracts: (dog or dogs or canine or canines).mp. (hip and (dysplasia or subluxation)).mp. ((total hip and (replacement or arthroplasty)) or THR or THA).mp. ((femoral head and (excision or osteotomy or ostectomy)) or excision arthroplasty or FHO).mp. 1 and 2 and (3 or 4) PubMed: #1 dog or canine #2 hip and (dysplasia or subluxation) #3 ((total hip and (replacement or arthroplasty)) or THR or THA) #4 ((femoral head and (excision or osteotomy or ostectomy)) or excision arthroplasty or FHO) #5 #1 and #2 and (#3 or #4) The references of relevant articles were reviewed for further relevant articles missed in the initial search.
Dates searches performed:	08 Oct 2021

**Exclusion / Inclusion Criteria:** 

Exclusion:	Book chapters, conference proceedings, articles not available in English, clinical reviews, case studies, studies with fewer than 10 dogs at time of follow-up, studies using prototypic hip replacement models, biceps femoris muscle sling technique for FHO.
Inclusion:	Articles written in English relevant to the PICO question, studies with ≥ 6 months follow-up for all included cases, studies evaluating treatment primarily for hip dysplasia (hip dysplasia as the majority of cases included in the study), commercially available total hip replacement implants.

**Search Outcome:** 

Database	Number of results	Excluded – Reviews	Excluded – Not available in English	Excluded – Not relevant to PICO question	Total relevant papers
CAB Abstracts	139	26	30	76	6
PubMed	105	14	3	80	8
Reference Checking	6	0	0	4	2
Total relevant papers when duplicates removed					12

## Conflict of Interest

The authors declare no conflicts of interest.

## Disclaimer

Knowledge Summaries are a peer-reviewed article type which aims to answer a clinical question based on the best available current evidence. It does not override the responsibility of the practitioner. Informed decisions should be made by considering such factors as individual clinical expertise and judgement along with patient’s circumstances and owners’ values. Knowledge Summaries are a resource to help inform and any opinions expressed within the Knowledge Summaries are the author’s own and do not necessarily reflect the view of the RCVS Knowledge. Authors are responsible for the accuracy of the content. While the Editor and Publisher believe that all content herein are in accord with current recommendations and practice at the time of publication, they accept no legal responsibility for any errors or omissions, and make no warranty, express or implied, with respect to material contained within. For further information please refer to our Terms of Use.

## References

[BIBR-1] Bayer K., Matiasovic M., Steger H., Böttcher P. (2019). Complications and Long-Term Outcome in 16 Canine Cementless Hybrid Hip Arthroplasties. Veterinary and Comparative Orthopaedics and.

[BIBR-2] Bergh M.S., Budsberg S.C. (2014). A Systematic Review of the Literature Describing the Efficacy of Surgical Treatments for Canine Hip Dysplasia (1948–2012. Veterinary.

[BIBR-3] Budsberg S.C., Chambers J.N., Lue S.L.van, Foutz T.L., Reece L. (1996). Prospective evaluation of ground reaction forces in dogs undergoing unilateral total hip replacement. American Journal of Veterinary Research.

[BIBR-4] Dueland R., Bartel D.L., Antonson E. (1977). Force-plant technique for canine gait analysis of total hip and excision arthroplasty [proceedings. Journal of the American Animal Hospital Association.

[BIBR-5] Duff R., Campbell J.R. (1977). Long term results of excision arthroplasty of the canine hip. The Veterinary Record.

[BIBR-6] Fitzpatrick N., Law A.Y., Bielecki M., Girling S. (2014). Cementless Total Hip Replacement in 20 Juveniles Using BFXTM Arthroplasty. Veterinary.

[BIBR-7] Ganesh T.N., Bridglalsingh Siobhan, Legall C. (2017). Femoral head and neck excision arthroplasty in 38 dogs: a two-year study with special reference to surgical indication and outcome. Indian Journal of Veterinary Surgery.

[BIBR-8] Gemmill T.J., Pink J., Renwick A., Oxley B., Downes C., Roch S., McKee W.M. (2011). Hybrid Cemented/Cementless Total Hip Replacement in Dogs: Seventy-Eight Consecutive Joint Replacements. Veterinary.

[BIBR-9] Guerrero T.G., Montavon P.M. (2009). Zurich Cementless Total Hip Replacement: Retrospective Evaluation of 2nd Generation Implants in 60 Dogs. Veterinary.

[BIBR-10] Massat B.J., Vasseur P.B. (1994). Clinical and radiographic results of total hip arthroplasty in dogs: 96 cases (1986–1992. Journal of the American Veterinary Medical Association.

[BIBR-11] Off W., Matis U. (2010). Excision arthroplasty of the hip joint in dogs and cats. Clinical, radiographic, and gait analysis findings from the Department of Surgery, Veterinary Faculty of the Ludwig-Maximilians-University of Munich. Veterinary and Comparative Orthopaedics and.

[BIBR-12] Olmstead M.L., Hohn R.B., Turner T.M. (1983). A five-year study of 221 total hip replacements in the dog. Journal of the American Veterinary Medical Association.

[BIBR-13] Parker R.B., Spencer C.P., Bloomberg M.S., Bitetto W., Rodkey W.G. (1984). Canine total hip arthroplasty: a radiographic correlation of clinical results in 20 cases. Journal of the American Animal Hospital Association.

[BIBR-14] Rawson E.A., Aronsohn M.G., Burk R.L. (2005). Simultaneous Bilateral Femoral Head and Neck Ostectomy for the Treatment of Canine Hip Dysplasia. Journal of the American Animal Hospital Association.

[BIBR-15] Vezzoni L., Vezzoni A., Boudrieau R.J. (2015). Long-Term Outcome of Zürich Cementless Total Hip Arthroplasty in 439 Cases. Veterinary Surgery.

